# Single centre retrospective review of plasma branched-chain amino acid levels in children with urea cycle disorders: Impact of treatment modalities and disease severity

**DOI:** 10.1016/j.ymgmr.2025.101190

**Published:** 2025-01-17

**Authors:** Mildrid Yeo, Preeya Rehsi, Jie Ming Yeo, Marjorie Dixon, Anupam Chakrapani

**Affiliations:** a Department of Paediatric Inherited Metabolic Disease, Great Ormond Street Hospital for Children, NHS Foundation Trust and Institute for Child Health, London, UK; b Dietetics, Great Ormond Street Hospital for Children, NHS Foundation Trust and Institute for Child Health, London, UK

**Keywords:** Urea cycle disorders, Branched chain amino acids, Nitrogen scavengers, Sodium benzoate, Sodium phenylbutyrate, Glycerol phenylbutyrate

## Abstract

Branched-chain amino acids (BCAAs) are important for normal growth, development, and function. In urea cycle disorders (UCDs), plasma BCAA levels can be relatively low; this has been attributed variously to low protein intake, hyperammonaemia, and nitrogen scavenger treatment. We undertook a retrospective review of plasma BCAA levels in individuals with UCDs comprising ornithine carbamoyltransferase deficiency (OTCD *n* = 22), arginosuccinate lyase deficiency (ASLD *n* = 12), and argininosuccinate synthase deficiency (ASSD *n* = 6). Scavenger treatment groups comprised sodium benzoate (NaBz, *n* = 20), sodium phenylbutyrate (NaPBA, *n* = 5), NaBz+NaPBA (*n* = 14), and a control group receiving neither NaBz nor NaPBA (n = 14). In these treatment groups, respectively, median (IQR) plasma levels of leucine were 54 (32), 55 (25), 58 (19), and 91 (70) μmol/L (leucine was lower in the NaBz group than the control, *p* = 0.0282) and numbers of individuals (%) with leucine below normal were 10/20 (50 %), 1/4 (25 %), 10/14 (71 %), and 2/9 (20 %). The pattern was similar for isoleucine and valine. In the NaBz group, plasma BCAA levels were inversely correlated with protein intake (*p* ≤ 0.01 to *p* ≤ 0.001), plasma ammonia level (p ≤ 0.01 to *p* ≤ 0.0001), and scavenger dose (p ≤ 0.0001). We speculate that individuals with greater disease severity may be prone to BCAA deficiency, caused by BCAA consumption when alternative urea disposal pathways are used. Practical reflections on our audit were that to increase the proportion of plasma BCAA levels in the normal range, we needed to alter the biological value of protein intake, prescribe higher doses of scavenger to facilitate safe levels of protein intake, and give EAA supplements if indicated.

## Highlights

• BCAAs are metabolised when alternate pathways for nitrogen disposal are used.
• This may explain why plasma BCAA levels can be relatively low in patients with UCDs.
• We speculate whether disease severity might determine BCAA levels in UCD patients.
• Some individuals need higher doses of scavenger to facilitate a safe level of protein.
• Where indicated, EAA supplements rich in BCAAs may also be needed.

## 1 Introduction

Urea cycle disorders (UCDs) are genetic defects that impair the body's ability to detoxify ammonia, a byproduct of protein metabolism, leading to potentially life-threatening hyperammonaemia. Individuals prone to hyperammonaemia are managed using protein-restricted diets that aim to provide at least the safe amount of protein, nitrogen, and indispensable (essential) amino acids (EAAs) to meet the body's requirements for growth and metabolic stability [1].

Of the EAAs, the branched chain amino acids (BCAAs) leucine, isoleucine, and valine are thought to be particularly important for normal growth, development, and function [2,3]. BCAA deficiency in individuals with UCDs have been attributed variously to low dietary intake [4], hyperammonaemia [5,6], and nitrogen scavenger treatment [7,8], although the exact mechanisms have not been determined definitively.

This retrospective review, undertaken as part of a clinical audit, considers these suggested causes of BCAA deficiency and examines their relationship with plasma BCAA levels in our UCD cohort. The results are intended to add to the literature on BCAAs levels in individuals with UCDs and to inform future research on this topic.

## 2 Materials and methods

### 2.1 Design

This was a retrospective single-centre audit undertaken at our specialist paediatric metabolic centre at Great Ormond Street Hospital for Children. Retrospective data spanning April 2000 to November 2018 were collated between September 2018 and March 2019. Data were obtained from an electronic document management (EDM) system. Individuals were included if they had a diagnosis of either argininosuccinate lyase deficiency (ASLD), ornithine transcarbamylase deficiency (OTCD), or argininosuccinate synthase deficiency (ASSD). These three groups were chosen as they represented the majority of our UCD cases. All individuals were prescribed around safe protein intake for age [3]. Exclusion criteria included liver transplant, active clinical trial participation, and absence of data on the EDM system. Data were depersonalised, which involved removing specific personal identifiers including name, NHS number, and date of birth, and the allocation of an audit reference number prior to analysis. Approval for the audit was given by Great Ormond Street Hospital's Audit Department. In line with hospital policy, ethical approval was not required.

### 2.2 Variables

Data collected included 1) individual UCD diagnosis and if made by biochemical or genetic testing, 2) plasma levels of all BCAAs, 3) plasma levels of two essential amino acids, phenylalanine and lysine, to provide a representation of overall essential amino acid levels, 4) plasma levels of ammonia and glutamine, 5) estimated protein intake based on individual recall, 6) type and amount of EAA supplement, and 7) nitrogen scavenger treatment and dosage. The data collection period preceded our use of the nitrogen scavenger glycerol phenylbutyrate (GPB), therefore the scavenger treatment analysis was limited to sodium benzoate (NaBz), sodium phenylbutyrate (NaPBA), a combination of NaBz+NaPBA, and a control group treated with neither NaBz nor NaPBA.

### 2.3 Laboratory analysis

Blood samples for laboratory measurements were taken at routine hospital out-patient appointments where patients were not reported to be unwell in clinic letters. Samples were therefore not controlled and could reflect the fed or fasted state. Plasma ammonia levels were measured by reflectance spectrophotometry at 600 nm after reaction with bromophenol blue indicator using the VITROS 5600 Integrated System (Ortho Clinical Diagnostics). Plasma amino acid levels were measured by absorption spectrometry at 570 nm of the ninhydrin derivative following protein precipitation with 5-sulphosalicylic acid containing a specified quantity of internal standard (S (2 Aminoethyl) L cysteine hydrochloride (AEC)). Separation by cation exchange chromatography was performed using the Biochrom 30+ Amino Acid Analyser Physiological System (Biochrom Limited). Normal reference ranges were: glutamine 530–960, phenylalanine 42–182, lysine 114–316, leucine 46–230, isoleucine 27–105, and valine 80–370, all μmol/L.

### 2.4 Statistical analysis

Statistical analysis was performed using GraphPad Prism 9. A *p*-value of ≤0.05 was considered statistically significant. For regression analysis of the relationships of BCAA levels with other variables, data were analysed using Spearman's rank for correlation, and all BCAA measurements were included rather than a single mean or median for each patient, because within-patient relationships were of relevance. Regression analysis was done by scavenger treatment group to enable any differences in relationships within those groups to be discerned. For comparison of BCAA levels between groups, data comprised single medians for each patient and were analysed using Kruskal-Wallis test with Dunn's multiple comparisons. Data were presented as medians and interquartile ranges. Additional sub-analyses compared the biochemical data between groups and analysed correlations between variables using the same statistical methods as for the whole cohort analysis.

## 3 Results

### 3.1 Demographics

Forty-three individuals with UCDs were identified with a total of 504 clinic letters. Three individuals were later excluded due to absence of clinic letters leaving 40 individuals in the cohort, 55 % (22/40) male and 45 % (18/40) female. Diagnosis of a UCD was determined by biochemical or genetic testing (Table 1). Age at diagnosis of a UCD was pre-natal via family history in 25 % (10/40), in the neonatal period in 33 % (13/40), beyond the neonatal period in 40 % (16/40), and there were no details for one individual (1/40, 3 %). Some individuals appear in Table 1 more than once, due to being on multiple nitrogen scavenger treatment regimens in the data collection period.

**Table 1:** UCD type, method of diagnosis, and nitrogen scavenger treatment.

UCD type (number of individuals)	Method of diagnosis		Number of individuals receiving each scavenger treatment†				
	Biochemical	Genetic	No scavenger	NaBz only	NaPBA only	NaBz+NaPBA	Total
ASLD⁎ (n = 12)	9	3	2	9	2	3	16
OTCD⁎⁎ (*n* = 22)	1	21	10	9	3	8	30
ASSD⁎⁎⁎ (n = 6)	6	0	2	2	0	3	7
Total (*n* = 40)	16	24	14	20	5	14	53

† A total of 13 individuals received more than one treatment regimen over the course of the audit period.

⁎ ASLD included two on NaBz initially then later had NaPBA added as dual scavenger therapy, one who switched from NaBz to NaPBA, and one who had no scavenger initially, then later treated with NaBz.

⁎⁎ OTCD included two who were on NaBz initially who later had NaPBA added as dual scavenger therapy, one who had no scavenger initially then started treatment with NaBz+NaPBA, one who switched from NaBz+NaPBA to NaBz, two who switched from NaBz+NaPBA to NaPBA, and two who were on NaPBA initially who later had NaBz added as dual scavenger therapy. Nine OTCD were female carriers.

⁎⁎⁎ ASSD included one on NaBz initially who later stopped the scavenger.

### 3.2 Relationship between BCAA levels and estimated protein intake

**Fig. 1:**
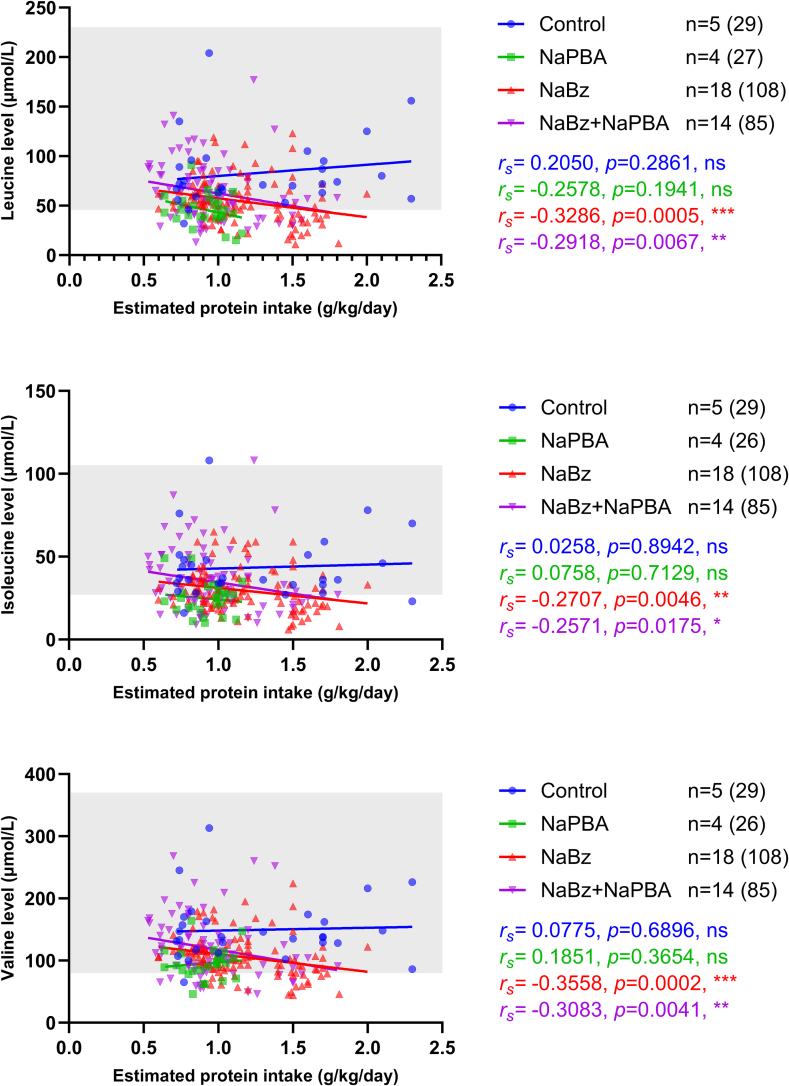
BCAA levels (μmol/L) and estimated protein intake (g/kg/day) by treatment group. Shading indicates the normal reference range; *r*_*s*_ indicates the Spearman's rank correlation coefficient.

Statistically significant inverse correlations were observed between protein intake (g/kg/day) and plasma levels of each BCAA in individuals receiving NaBz and NaBz+NaPBA (Fig. 1). Daily protein intake and plasma essential amino acids in the control group appeared higher than in each of the scavenger treatment groups but the difference was not statistically significant (Supplementary Table 1). Eight individuals (8/40, 20 %) received EAA supplementation during the period assessed. These individuals had typically failed to reach adequate levels of plasma amino acids through natural dietary protein and were given EAA supplements to try to normalise their levels. Of the BCAA measurements assessed, 128 (128/1258, 10 %) were during a period when an individual was receiving an EAA supplement, by treatment group, 0/162 (0 %) controls, 6/115 (5 %) NaPBA, 6/525 (1 %) NaBz, and 116/456 (25 %) NaBz+NaPBA.

### 3.3 Relationship between BCAA levels and ammonia levels

**Fig. 2:**
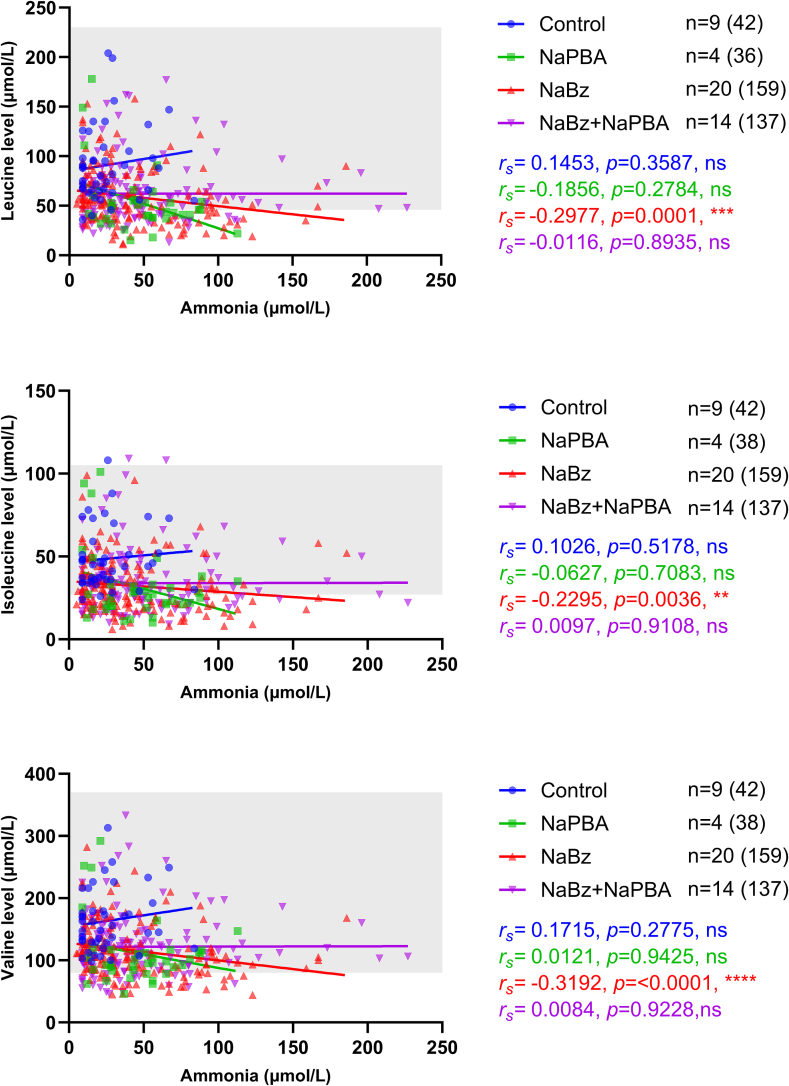
BCAA levels (μmol/L) and ammonia levels (μmol/L) by treatment group. Shading indicates the normal reference range; *r*_*s*_ indicates the Spearman's rank correlation coefficient.

There were inverse correlations between plasma ammonia levels and plasma levels of each BCAA in individuals receiving NaBz (Fig. 2).

### 3.4 Relationship between BCAA levels and nitrogen scavenger dose

**Fig. 3:**
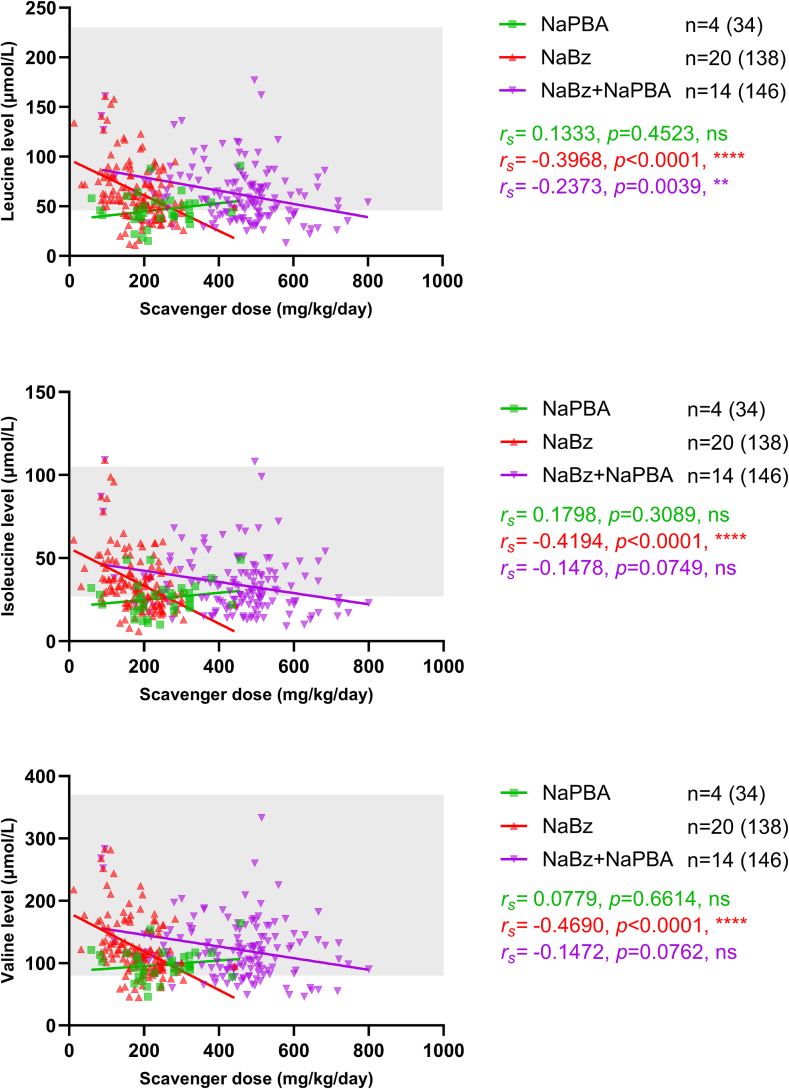
BCAA levels (μmol/L) by scavenger dose (mg/kg/day). Shading indicates the normal reference range; *r*_*s*_ indicates the Spearman's rank correlation coefficient.

There were inverse correlations between scavenger dose and plasma levels of each BCAA in individuals receiving NaBz (Fig. 3). The median dose (IQR) of nitrogen scavenger treatment in the NaPBA and NaBz groups was 252 (239) and 201 (79) mg/kg/day. In the NaBz+NaPBA group, the median combined dose (IQR) was 449 (189) mg/kg/day, and when separated into NaPBA and NaBz doses within the group, it was 225 (83) and 220 (65) mg/kg/day respectively.

### 3.5 Amino acid levels by type of nitrogen scavenger treatment

The control group appeared to have higher levels of BCAAs and lower levels of glutamine versus each of the scavenger treatment groups, but the differences were not statistically significant, nor were any differences discernible between the groups in phenylalanine nor lysine (Supplementary Table 1). The only exception was for the plasma leucine level, which was lower in the NaBz group than the control group (54 versus 91 μmol/L, *p* = 0.0282). The number of individuals with a leucine measurement below the normal range in the groups were: control 2/9 (20 %), NaPBA 1/4 (25 %), NaBz 10/20 (50 %), and NaBz+NaPBA 10/14 (71 %); a similar pattern was observed between the control and scavenger groups for isoleucine and valine (Supplementary Table 2).

## 4 Discussion

Levels of plasma BCAAs in our cohort varied. While over two-thirds of measurements were within the normal range, almost one-third (362/1258, 29 %) were below the lower limit of normal; the low levels were almost exclusively in those with disease severe enough to warrant a nitrogen scavenger. Use of EAA supplements was uncommon, with only eight individuals (8/40, 20 %) receiving a supplement; these individuals were mostly receiving two nitrogen scavengers. Current strategies to normalise levels of plasma BCAAs include 1) trying to ensure individuals achieve an adequate intake of BCAA from natural protein as the first priority, and 2) introducing an EAA supplement if there are other indications that it may be appropriate, such as slow growth rate or low protein quality. Where natural protein intake is insufficient, EAA supplements may be considered if an individual is deficient in several of these key amino acids [3]. However, evidence from the literature suggests that while protein intake and use of EAA supplements can increase or maintain BCAA levels in plasma, other factors appear to cause a reduction in plasma BCAA levels [9,10]. Hyperammonaemia and nitrogen scavenger treatment have been reported to reduce plasma BCAA levels, therefore a strategy to achieve normal levels of plasma BCAAs should seemingly include not just adequate protein and BCAA intake, but also tight control of ammonia using - ideally - a nitrogen scavenger that does not deplete BCAAs.

### 4.1 Dietary protein as a means of achieving normal levels of BCAAs

In our audit, protein intake (g/kg) in individuals receiving NaBz as monotherapy was inversely correlated with levels of BCAAs. A theoretical explanation for this finding might be that the increase in protein intake was associated with an increase in ammonia production, and this in turn caused a reduction in BCAA levels. Such an effect of ammonia on BCAAs has been reported previously [9,10], and our data did show an inverse relationship between ammonia level and BCAA levels, again albeit within the NaBz group only.

Whilst natural protein and EAA supplements, if indicated, are the current approach to achieving normal levels of plasma BCAAs, it has been suggested that dietary EAA supplements cannot reliably increase levels of plasma BCAA in UCD individuals. In a study of 307 individuals longitudinally followed by the Urea Cycle Disorders Consortium (UCDC) and the European registry and network for Intoxication type Metabolic Diseases (*E*-IMD), levels of BCAAs in the population were within the lower normal range or reduced, even in individuals receiving EAA supplements, leading the authors to conclude that prescribing EAA supplements was not sufficiently compensating for BCAA depletion in individuals with UCDs [10]. However, this was a retrospective study, not a randomised controlled trial, and it might be expected that individuals receiving EAA supplements perhaps had a more severe phenotype and were subject to greater protein restriction than those not receiving supplements. If individuals in the supplement group had not received supplements, it is very possible their levels of BCAAs would have been lower than the comparator group. As it was, they did receive supplements, and their levels of BCAAs matched those of the group that did not receive EAA supplements, which might be regarded as a good outcome. This more optimistic outlook was reflected in the interpretation of results from another multicentre European registry study of 361 UCD individuals. This study reported that UCD individuals who received amino acid mixtures supplemented with EAA, and accordingly a lower natural protein prescription, achieved plasma isoleucine and valine levels and isoleucine:leucine:valine ratios similar to individuals not receiving amino acid mixtures. This was regarded as suggesting that EAA supplements can have a beneficial effect in individuals with UCDs in the stable disease period [4]. Indeed, EAA supplements as part of the total daily protein intake may be beneficial because, theoretically, by limiting the intake of non-EAA, waste nitrogen is utilised to synthesise these non-EAAs and hence nitrogen destined for excretion as urea will be reduced. This, however, has never been evidenced in clinical studies and is generally not routine practice [1]. Of note, EAA supplements have limitations; they are often unpleasant-tasting and lack the additional nutrients provided by natural protein sources [3].

Regardless of any doubts about the effectiveness of EAA supplements to increase BCAA levels, the most recent European guidelines on UCD management state that EAA supplements rich in BCAA are essential when individuals are not receiving adequate EAA intake from natural foods and supplements, although it should be emphasised that optimising natural protein is to be addressed first [3]. Notably, in protein-restricted diets, the choice between high biological value (HBV) and low biological value (LBV) protein sources is significant. HBV proteins provide a more complete profile of indispensable amino acids, but LBV proteins often offer greater dietary variety and energy per gram of protein. Care must be taken to ensure that restricted diets containing predominantly LBV proteins do not lead to deficiencies in one or more indispensable amino acids.

In our cohort, protein intake was not associated reliably with levels of plasma BCAAs, even in the control group, indicating that factors other than overall protein intake need to be considered to enable plasma BCAA levels to be maintained in the normal range.

### 4.2 Tight control of ammonia as a means to achieving normal levels of BCAAs

**Fig. 4:**
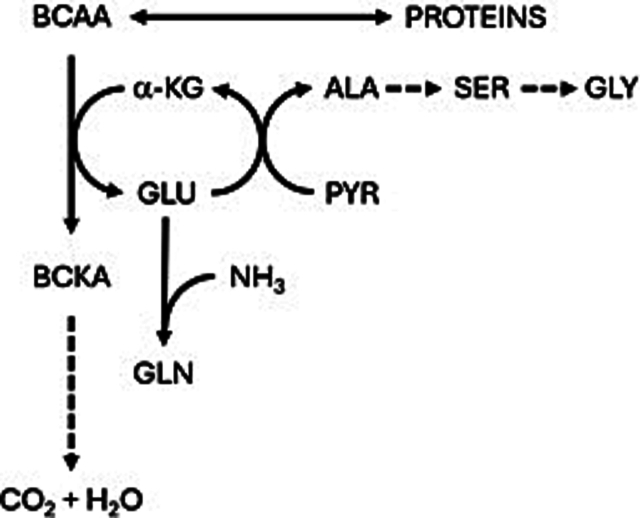
Simple representation of production of glutamine and alanine in muscle tissue Adapted from [5]. ALA, alanine; BCAA, branched-chain amino acids; BCKA, branched-chain keto acids. GLU, glutamate; GLN, glutamine; GLY, glycine; PYR, pyruvate; SER, serine; α-KG, α-ketoglutarate.

Hyperammonaemia has been proposed as a cause of low BCAA levels in UCDs [9]. Elevated ammonia drives the production of glutamine and alanine - the major non-toxic interorgan ammonia carriers – via a process which occurs in skeletal muscle involving deamination of BCAAs to their branched chain ketoacid (BCKA) equivalents during the production of glutamate (Fig. 4) [11,12]. In this manner, BCAAs are thought to be metabolised at an increased rate when ammonia is high, and this is reflected in a decrease in levels of BCAAs in plasma. It has been estimated that BCAAs may contribute a minimum of 60 % of the nitrogen required for alanine synthesis [13]. Therefore, it is reasonable that an increase in plasma ammonia, prompting an increase in glutamine and alanine synthesis, would be accompanied by a reduction in levels of BCAAs in plasma.

Achieving tight control of ammonia in UCD individuals with more severe phenotypes relies on an adequate dose of one or more nitrogen scavengers. However, it is important to note that scavenger treatment does not affect ammonia production; the scavengers simply bind nitrogen that is produced when the ammonia is metabolised. Thus, regardless of scavenger type and dose, ammonia will continue to be generated and metabolised via mechanisms that involve BCAA catabolism. A logical inference is that depletion of BCAAs is therefore a function of UCD severity, likely more so than scavenger type or dose. Individuals with a severe phenotype and poor ammonia control, manifested as elevated plasma ammonia, are likely to need to rely heavily on alternative pathways for ammonia detoxification, involving significant deamination of BCAAs. Indeed, in a registry study involving 307 UCD individuals, disease severity was found to be associated with low levels of BCAAs [10]. However, if severity of phenotype determines BCAA levels, it poses the question of why in our data we also observed an inverse relationship between levels of all three BCAAs and NaBz dose and levels of leucine and NaBz+NaPBA dose. It may be related to the likelihood of disease severity determining the degree of ammonia control, and the degree of ammonia control in turn determining scavenger dose, i.e. patients with greater disease severity require a higher dose of scavenger to achieve ammonia control. As such, the more severe the disease, the greater the production of ammonia, the greater the consumption of BCAAs to channel the ammonia via alternate pathways, and the higher the scavenger dose required to remove the ammonia being produced. This could explain why scavenger dose and plasma BCAA levels are inversely related. This contention that disease severity is a determinant of levels of BCAAs could be explored further by measuring residual enzymatic activities and investigating their relationship with plasma BCAA levels.

Another approach to avoiding BCAA depletion via ammonia control may be to target a reduction in ammonia production, rather than scavenging ammonia after it has been produced. However, the treatments available, such as antibiotics or lactulose, are not always effective [14]. An effective treatment is liver transplant. According to data from the UCDC and the *E*-IMD, 19 individuals who received a transplant at a mean age of 2.2 years (range 0.4 to 7.8 years) achieved levels of BCAAs well within the normal range after transplant, compared to reduced or low normal values before transplant, indicating that the functionality of the urea cycle had been restored and there was no longer a need for reliance on the alternative pathways with their associated BCAA metabolism [10]. Those options aside, achieving tight ammonia control using nitrogen scavengers remains the priority, to avoid hyperammonaemic crises and minimise the time spent with elevated ammonia levels. Even mildly elevated ammonia levels, or covert hyperammonaemia, can still have injurious effects on the brain and organs [15]. Once tight ammonia control is achieved, the diet can be manipulated to try to achieve a safe level of natural protein and an EAA supplement introduced if warranted.

### 4.3 Choice of scavenger as a means to achieving normal levels of BCAAs

In our audit, individuals who were not receiving nitrogen scavengers tended to have higher levels of BCAAs and presumably had less severe phenotypes than individuals who were receiving scavenger treatment. However, it is not clear whether the lower BCAA levels in individuals receiving scavengers reflects a true independent effect of scavenger treatment or simply reflects the likelihood that individuals receiving scavenger treatment will have worse disease severity than those not receiving treatment. In one of the larger studies reported in the literature, the Longitudinal Study of Urea Cycle Disorders, a collaborative multi-centre study of the Urea Cycle Disorders Consortium, BCAA levels were investigated in 553 paediatric and adult UCD individuals. Individuals receiving NaPBA had lower levels of BCAAs compared to individuals not receiving NaPBA, although there were differences between the groups in baseline characteristics: individuals receiving NaPBA were younger, more likely to have neonatal-onset disease, and comprised a lower percentage of female individuals with ornithine transcarbamylase deficiency (OTCD). When these variables were accounted for, a difference in BCAA levels between groups remained, although no evidence was found of a correlation between dosage of NaPBA and levels of BCAAs [8]. Individuals receiving NaBz were also found to have lower levels of BCAAs compared to individuals not receiving NaBz. However, individuals receiving NaBz also had lower levels of prealbumin, indicative of a low protein intake, which the investigators suggested may have caused the low levels of BCAAs observed [7]. Evidence in the literature regarding the associations between scavengers and plasma BCAA levels in UCD individuals tends to be confounded by various factors, meaning the exact relationship between scavengers and plasma BCAA levels remains unclear.

**Fig. 5:**
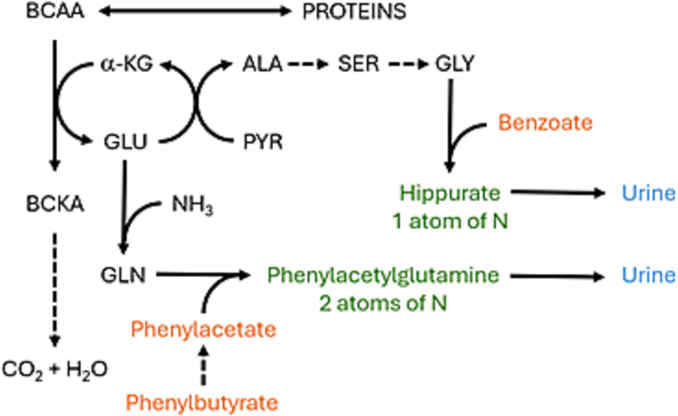
Simple representation of scavenging action of benzoate and phenylbutyrate. ALA, alanine; BCAA, branched-chain amino acids; BCKA, branched-chain keto acids. GLU, glutamate; GLN, glutamine; GLY, glycine; N, nitrogen; PYR, pyruvate; SER, serine; α-KG, α-ketoglutarate.

There are some theoretical considerations regarding whether NaBz or NaPBA might have the greatest effect on BCAA levels, but their practical significance is not clear. The simplified model below (Fig. 5) represents the metabolism of BCAAs to produce the major ammonia sinks glutamine and alanine, along with a depiction of the scavenging moieties of NaBz and NaPBA, which are benzoate and phenylacetate respectively.

As indicated, benzoate conjugates glycine to form hippurate, which is excreted in urine. More glycine is synthesized from serine which, in turn, is replaced using nitrogen derived from alanine or glutamine, meaning each mole of benzoate administered should remove one mole of waste nitrogen [16]. Phenylacetate conjugates glutamine to form phenylacetateglutamine, which is excreted in urine. More glutamine is synthesized from glutamate, meaning each mole of phenylacetate should remove two moles of waste nitrogen. Thus, NaPBA, in theory, scavenges twice as much nitrogen as NaBz, suggesting that NaPBA should be a more economical scavenger in terms of ammonia detoxified per BCAA consumed. Further data is needed to explore whether the theory is reflected in practice.

In our cohort, we did not observe a statistically significant difference between NaBz and NaPBA in their effects on BCAA levels nor ammonia detoxification; however, the small size of the NaPBA group likely limited the statistical power to detect differences versus other treatment groups. We previously reported a case series of 20 paediatric UCD individuals transitioned from NaBz to glycerol phenylbutyrate (GPB) at our centre. Ammonia control improved after the transition but there was no increase in BCAA levels [17]. Importantly, there was no decrease either, suggesting that GPB was more BCAA-sparing i.e. GPB appeared able to maintain the same level of BCAAs as NaBz whilst maintaining a lower ammonia level. Mean BCAA levels remained within the normal range. Data from recent controlled clinical studies with GPB provide a substantial amount of data on BCAA levels, in contrast to the paucity of data from clinical studies with NaBz and NaPBA. The studies with GPB have indicated that in a controlled setting, using the doses defined in the Summary of Product Characteristics, it is possible to control ammonia and achieve normal levels of BCAAs [18,19]. GPB is a slower release scavenger than NaBz and NaPBA; it has a longer duration of scavenging action, and it can achieve tighter ammonia control. How or whether this characteristic of GPB confers a BCAA-sparing effect is not known. A topic for further investigation may be whether maintaining tight ammonia control using GPB can reduce the amount of ammonia available to be recycled in further reactions that involve metabolism of BCAAs. A multi-centre study in Spain involving 48 paediatric UCD individuals transitioned to GPB reported mean BCAA levels within the normal range in individuals receiving GPB. However, the investigators suggested that the levels of BCAAs achieved might be related to EAA supplementation, with 60 % of individuals receiving 30 % of their proteins as EAAs. This was suggested to increase the intake of BCAAs, since EEAs are rich in BCAAs, and decrease the ammonia load in the urea cycle, since EAAs contain less nitrogen than natural proteins [18].

### 4.4 Audit limitations

We acknowledge the limitations of our audit. Data were collected retrospectively from available clinic letters and chemical pathology reports, and individuals were assumed to have complied with their prescribed diets and treatment plans. The biochemistry samples were uncontrolled, which may explain why a small number of the ammonia measurements appear high considering these were from samples from supposedly well controlled individuals attending routine appointments. The amino acid analysis did not include alanine, glycine and serine, which may have been helpful, given the involvement of these amino acids in ammonia detoxification and BCAA metabolism. Our data reflect the population at our centre during the analysis period, resulting in uneven group sizes. Smaller groups, particularly the NaPBA treatment group, likely limited the ability to discern statistically significant relationships within groups and differences between groups. Use of EAA supplements is likely to have resulted in elevated plasma BCAAs in some individuals, mostly those receiving NaBz+NaPBA, which will have attenuated the power of the BCAA regression analysis and between-group comparisons for that treatment group. The OTCD group included female carriers (9/22, 41 %) but this did not skew the trends observed in the overall cohort. Whilst overall, our cohort size is larger than some of the reports in the literature, the statistical power is still limited.

## 5 Conclusion

Our data show inverse associations of plasma BCAA levels with dietary protein, ammonia exposure, and scavenger dose. We speculate that factors previously suggested to cause low plasma BCAAs – including high plasma ammonia levels and the use of nitrogen scavengers - may actually be surrogate markers of disease severity. The relationships identified between the variables we measured may not be true cause and effect. Instead, disease severity and the corresponding extent to which the alternative pathways are used may be the primary determinant of plasma BCAA levels. This would suggest that individuals with greater disease severity may be more prone to BCAA deficiency. If so, such individuals may also be more likely to require higher scavenger doses to control ammonia, and these higher doses may be needed to enable adequate intakes of natural dietary protein, with EAA supplements where indicated, to maintain plasma BCAA levels in the normal range. We previously described changing scavenger treatment from NaBz to GPB in 20 individuals with UCDs at our centre which achieved a greater reduction in plasma ammonia without any reduction in plasma BCAAs. Other studies have found that GPB can control ammonia and maintain BCAAs in the normal range, with supplements where appropriate, therefore strategies do seem to be available for preserving BCAA levels. Further research is required to elucidate definitively the mechanisms by which BCAAs are depleted in individuals with UCDs. Practical reflections on our audit were that to increase the proportion of plasma BCAA levels in the normal range, we needed to alter the biological value of protein intake, prescribe higher doses of scavenger to facilitate safe levels of protein intake, and give EAA supplements if indicated.

## Informed consent statement

Ethical approval and informed consent for being included in the audit were not required.

## CRediT authorship contribution statement

**Mildrid Yeo:** Writing – original draft, Validation, Resources, Investigation, Formal analysis, Data curation, Conceptualization. **Preeya Rehsi:** Writing – review & editing, Resources, Investigation. **Jie Ming Yeo:** Writing – review & editing, Resources, Investigation, Formal analysis, Data curation. **Marjorie Dixon:** Writing – review & editing, Supervision, Resources, Investigation. **Anupam Chakrapani:** Writing – review & editing, Supervision, Resources, Investigation.

## Declaration of competing interest

Mildrid Yeo and Marjorie Dixon have received speaker honoraria from Immedica. The remaining authors declare no conflicts of interest.

## Data Availability

Data can be made available upon reasonable request to the corresponding author (MY). The data are not publicly available due to the sensitivity of individual data.

## Appendix A Supplementary data

**Supplementary Table 1**. Protein intake, EAA supplementation, and plasma essential amino acid levels by scavenger treatment Image 1

**Supplementary Table 2**. Plasma BCAA measurements below the normal range Image 2
