# Supplementary material for: Single centre retrospective review of plasma branched-chain amino acid levels in children with urea cycle disorders: Impact of treatment modalities and disease severity
Source: Mol Genet Metab Rep. 2025 Jan 17;42:101190. doi: 10.1016/j.ymgmr.2025.101190 (PMC11786853; doi:10.1016/j.ymgmr.2025.101190)
Supplement: Supplementary file 1 — Supplementary Table 1. Protein intake, EAA supplementation, and plasma essential amino acid levels by scavenger treatment [file mmc1.docx]

**Single Centre Retrospective Review of Plasma Branched-Chain Amino Acid Levels in Children with Urea Cycle Disorders: Impact of Treatment Modalities and Disease Severity**

**Mildrid Yeo****,^a^* Preeya Rehsi,^a^ Jie Ming Yeo,^a^ Marjorie Dixon,^b^ Anupam Chakrapani^a^**

^a^ Department of Paediatric Inherited Metabolic Disease, Great Ormond Street Hospital for Children, NHS Foundation Trust and Institute for Child Health, London, UK, **^b^** Dietetics, Great Ormond Street Hospital for Children, NHS Foundation Trust and Institute for Child Health, London, UK

**Supplementary Table 1.** Protein intake, EAA supplementation, and plasma essential amino acid levels by scavenger treatment

|  | **Treatment group** | | | |
| --- | --- | --- | --- | --- |
|  | **Control** | **NaPBA** | **NaBz** | **NaPBA +NaBz** |
| Estimated protein intake (g/kg/day) median (IQR), n | 1.6  (1.1), 5 | 0.9  (0.4), 4 | 1.1  (0.3), 18 | 0.9  (0.3), 14 |
| Number (%) of BCAA measurements taken during period of EAA supplementation | 0/162 (0%) | 6/115 (5%) | 6/525 (1%) | 116/456 (25%) |
| Phenylalanine (µmol/L), median (IQR), n | 48  (12.5), 9 | 43  (16.7), 4 | 44  (7.4), 20 | 39  (15.4), 14 |
| Lysine (µmol/L), median (IQR), n | 125  (82.0), 9 | 138  (67.3), 4 | 119  (50.1), 20 | 148  (65.3), 14 |
| Glutamine (µmol/L), median (IQR), n | 707  (169), 9 | 948  (618), 4 | 791  (287), 20 | 826  (378), 14 |
| Leucine (µmol/L), median (IQR), n | 91  (69.7), 9 | 55  (25.1), 4 | 54  (32.1), 20 | 58  (18.8), 14 |
| Isoleucine (µmol/L), median (IQR), n | 44  (34.7), 9 | 32  (13.3), 4 | 30  (17.1), 20 | 31  (15.0), 14 |
| Valine (µmol/L), median (IQR), n | 171  (98.2), 9 | 110  (52.6), 4 | 116  (69.5), 20 | 113  (34.7), 14 |

IQR: Interquartile range, distance between the 25th and 75th percentiles. There were no statistically significant differences between any of the treatment groups for any of the variables measured except for leucine, where the value for the control group was higher than the NaBz group (p=0.0282). Normal reference ranges were: phenylalanine 42-182, lysine 114-316, glutamine 530-960, leucine 46-230, isoleucine 27-105, and valine 80-370, all µmol/L.
