# Supplementary material for: Single centre retrospective review of plasma branched-chain amino acid levels in children with urea cycle disorders: Impact of treatment modalities and disease severity
Source: Mol Genet Metab Rep. 2025 Jan 17;42:101190. doi: 10.1016/j.ymgmr.2025.101190 (PMC11786853; doi:10.1016/j.ymgmr.2025.101190)
Supplement: Supplementary file 2 — Supplementary Table 2. Plasma BCAA measurements below the normal range [file mmc2.docx]

**Single Centre Retrospective Review of Plasma Branched-Chain Amino Acid Levels in Children with Urea Cycle Disorders: Impact of Treatment Modalities and Disease Severity**

**Mildrid Yeo****,^a^* Preeya Rehsi,^a^ Jie Ming Yeo,^a^ Marjorie Dixon,^b^ Anupam Chakrapani^a^**

^a^ Department of Paediatric Inherited Metabolic Disease, Great Ormond Street Hospital for Children, NHS Foundation Trust and Institute for Child Health, London, UK, **^b^** Dietetics, Great Ormond Street Hospital for Children, NHS Foundation Trust and Institute for Child Health, London, UK

**Supplementary Table 2.** Plasma BCAA measurements below the normal range

| **BCAA** | **Number (%) of individuals with at least one BCAA measurement below normal** | | | |
| --- | --- | --- | --- | --- |
|  | **Control** | **NaPBA** | **NaBz** | **NaBz+NaPBA** |
| Leucine | 2/9, 20% | 1/4, 25% | 10/20, 50% | 10/14, 71% |
| Isoleucine | 2/9, 20% | 3/4, 75% | 15/20, 75% | 12/14, 86% |
| Valine | 1/9, 10% | 2/4, 50% | 9/20, 45% | 6/14, 43% |
|  | **Number (%) of BCAA measurements below the normal range** | | | |
|  | **Control** | **NaPBA** | **NaBz** | **NaBz+NaPBA** |
| Leucine | 3/54, 6% | 18/39, 46% | 59/175, 34% | 48/152, 32% |
| Isoleucine | 4/54, 7% | 19/38, 50% | 77/175, 44% | 65/152, 43% |
| Valine | 1/54, 2% | 8/38, 21% | 32/175, 18% | 28/152, 18% |

Normal range reflects the reference range at our laboratory. Normal reference ranges were: leucine 46‑230, isoleucine 27‑105, and valine 80‑370, all µmol/L.
